# Comparison of Antimycotic Activity of Originator and Generics of Voriconazole and Anidulafungin against Clinical Isolates of *Candida albicans* and *Candida glabrata*

**DOI:** 10.3390/jof8020195

**Published:** 2022-02-17

**Authors:** Alina Karoline Nussbaumer-Pröll, Sabine Eberl, René Welte, Tiziana Gasperetti, Jana Marx, Romuald Bellmann, Markus Zeitlinger

**Affiliations:** 1 Department of Clinical Pharmacology, Medical University of Vienna, 1090 Vienna, Austria; alina.nussbaumer-proell@meduniwien.ac.at (A.K.N.-P.); sabine.eberl@meduniwien.ac.at (S.E.); 2 Department of Clinical Pharmacokinetics, Innsbruck Medical University, 6020 Innsbruck, Austria; rene.welte@i-med.ac.at (R.W.); tiziana.gasperetti@i-med.ac.at (T.G.); jana.marx@i-med.ac.at (J.M.); romuald.bellmann@i-med.ac.at (R.B.)

**Keywords:** antifungals, generic, innovator, MIC, TKC, *C. albicans*, *C. glabrata*

## Abstract

Background: Concerns have been expressed about the interchangeability of innovator and generic antifungals in their activity and chemical stability. Materials/methods: The activity of two different antimycotics was tested, each with one originator and two generics. For voriconazole, the originator VFEND^®^ (Pfizer) and the generics (Ratiopharm and Stada) were used for susceptibility testing (21 clinical isolates of *Candida albicans* (*C. albicans*); ATCC-90028 *C. albicans*) in RPMI growth media in compliance with the EUCAST criteria. Likewise, for anidulafungin, the originator ECALTA^®^ (Pfizer) and the generics (Stada and Pharmore) were used for testing (20 clinical isolates of *Candida glabrata* (*C. glabrata*); ATCC-22019 *Candida parapsilosis* (*C. parapsilosis*)). Time Kill Curves (TKC) with concentrations above and below the respective MIC were performed for one strain for each antifungal. Stability testing of the antimycotics stored at 4 °C and at room temperature over 24 h was done, and samples were subsequently analyzed with HPLC. Results: MIC results showed no significant difference in activity of generic and innovator antimycotic in all settings, which was also confirmed by TKC. Stability testing revealed no differences between originator and generic drugs. Conclusions: The present study demonstrates the interchangeability of generic and originator antimycotic in-vitro, potentially leading to broader public acceptance for generic antimycotics.

## 1. Introduction

Generic drugs become more and more available on the market, which helps reduce health care associated costs [1]. When two drugs contain the same active substance(s) (qualitatively and quantitatively) and demonstrate a comparable exposure, they are expected to show the same efficacy and safety in treatment. Thus, no or only a few further measures are taken before a generic drug is released on the market [2]. Nevertheless, public concerns have been expressed about the interchangeability of innovator and generic.

Therefore, studies have been conducted in different fields, e.g., antibacterial and antiviral agents, to pursue public doubts. Most of these studies compare the pharmacokinetic properties of the tested drugs to show the interchangeability of generic and originator. A recent study compared the pharmacokinetics of two hepatitis C direct-acting antivirals, sofosbuvir, and daclatasvir, from 5 different generic companies, which could confirm that the generic drugs were bioequivalent to the originator [3].

Contrarily, another pharmacokinetic study showed that one of two tested generic formulations of amoxicillin could not fulfill bioequivalence in healthy subjects compared to the brand leader product, in the case of Cmax, based on a single-dose pharmacokinetic assessment [4].

Nevertheless, therapeutical equivalence should not only require pharmaceutical and pharmacokinetic aspects but should also include pharmacodynamic parameters [5].

In a recent study, the pharmacokinetic and pharmacodynamic profiling of generic amphotericin B colloidal dispersion was evaluated in a rat model regarding invasive candidiasis that could provide data for optimizing dosing regimens and breakpoints for antifungals [6].

Although animal testing is essential in research, there is a consensus that it should be kept to a necessary minimum. The European Medicines Agency guideline (*Guideline on the principles of regulatory acceptance of 3Rs [replacement, reduction, refinement]*) published in 2016 takes the position of replacing animal studies with in vitro models [7]. Moreover, in vitro assays might be more sensitive to detecting differences in efficacy than testing in biological systems.

Thus, we set out to investigate stability as well as the activity and efficacy of antimycotic generics and innovator of voriconazole and anidulafungin against clinical fungal isolates in vitro.

## 2. Materials and Methods

### 2.1. Fungal Strains

Reference strains were obtained from the American Type Culture Collection (ATCC). Reference strains ATCC-90028 *C. albicans* and ATCC-2201920 *C. parapsilosis* were used. The 21 clinical isolates of *C. albicans,* as well as 20 clinical isolates of *C. glabrata,* were collected and provided by the microbiological department of the General Hospital in Vienna.

### 2.2. Antifungals

For susceptibility testing and pharmacodynamic experiments, one originator and two generics of a fungistatic and a fungicidal agent were tested. Voriconazole (fungistatic) originator VFEND^®^ from Pfizer (manufacturer: Fareva Amboise, Pocé-sur-Cisse, France) and generics from Ratiopharm (manufacturer Merckle GmbH, Blaubeuren, Germany) and Stada (manufacturer: Stada GmbH, Vienna, Austria), each using 200 mg powder for infusion, dissolved in aqua ad iniectabilia, were used. Anidulafungin (fungicidal), originator ECALTA^®^ from Pfizer (manufacturer: Pfizer Manufacturing GmbH, Puurs, Belgium) and generics from Stada (manufacturer: Actavis, Nerviano, Italy) and Pharmore (manufacturer: Lyocontract GmbH, Ilsenburg (Harz), Germany), each using 100 mg powder for infusion, dissolved in aqua ad iniectabilia, were provided for experiments.

### 2.3. Growth Media

RPMI-1640 (dry powder, adapted with MOPS and Glucose) from Sigma-Aldrich was used as media for MIC testing as described in the EUCAST Definitive Document EDef 7.1 [8]. Furthermore, for TKC experiments Sabouraud growth media DifcoTM (SAB) and Sabouraud agar plates (SAG) from Becton, Dickinson and Company (BD) were used.

### 2.4. Minimal Inhibitory Concentration (MIC)

For MIC testing of reference strains and clinical isolates, flat-bottomed 96-well plates (nominal capacity 300 µL) were used. A dilution series of antifungal stock solutions (1:100) was prepared using 2× RPMI media. Further, 100 µL of these solutions were filled in wells of columns 1–10, receiving 2× the final concentration. Wells 11–12 were filled with 2× RPMI media. At this point, the plates were sealed and stored at −20 °C until usage (maximum 1 week). For MIC testing plates were thawed and an inoculum of 1–5 × 10^6^ CFU/mL (0.5× McFarland) (adjusted in SAB) of an overnight culture of test strains grown on SAG plates was prepared. Further, 100 µL of the inoculum of the specific strain was added to the wells of columns 1–10 to obtain a 50% dilution of the antifungal concentration on the plate. Well 11 served as a growth control and was filled with 100 µL of inoculum. Well 12 served as media control and was filled with 100 µL of sterile water. MIC testing was performed in triplicate for all settings with voriconazole concentrations ranging from 2–0.0039 mg/L against 21 clinical isolates of *C. albicans* and reference strain ATCC-90028 *C. albicans*. With anidulafungin concentrations ranging from 1–0.002 mg/L were tested against 20 clinical isolates of *C. glabrata* and reference strain ATCC-22019 *Candida parapsilosis* (*C. parapsilosis*). MIC evaluation was done with a photometric microdilution plate reader. All steps complied with the EUCAST Definitive Document EDef 7.1 for MIC testing of antifungal agents [8].

### 2.5. Time Kill Curves (TKC)

Time Kill Curves (TKC) with concentrations above and below the respective MIC of one representative strain in SAB media were performed. Strains were selected by choosing one strain with constant MIC values (no change in MIC within triplicates) and good growth behavior.

In the voriconazole setting, the originator and the 2 generics were tested against ATCC-90028 *C. albicans* (MIC 0.015 mg/L) with concentrations 1×, 4×, and 16× MIC. In the anidulafungin setting originator and the 2 generics were tested against 722H *C. glabrata* (MIC 0.06 mg/L) with concentrations 1×, 0.5×, 0.25×, and 0.125× MIC.

In the voriconazole setting, 14-mL tubes were filled with 2700 µL SAB medium, and 300 µL inoculum with a 0.5× McFarland (adjusted in SAB) was added and incubated for 1 h at 37 °C. Before adding the antimycotic, 100 µL of the tubes were drawn, and subsequently 7 dilutions (with NaCl in 96-well plates) were dropped on SAG plates to evaluate the CFU/mL. After adding voriconazole, samples were further incubated in the 37 °C water bath. Subsequent samples were drawn at 7, 24, 48, and 72 h.

The same procedure was performed for anidulafungin until 0 h. Subsequent samples were drawn at 3, 7, and 24 h.

### 2.6. Evaluation of Stability of Antibiotics

Stability testing of the antimycotics was done with samples stored at 4 °C and room temperature (RT) over 48 h. Three aliquots (each 1 mL) of the antimycotic solutions (prepared as described above) that were stored at 4 °C and RT (21–25 °C) were taken at 0, 24, and 48 h. Samples were stored at −80 °C in 1.5 mL Eppendorf tubes until HPLC analysis was performed.

### 2.7. Chemical Analysis

#### 2.7.1. Chromatography for Voriconazole

HPLC was carried out on a Prominence Modular LC20 system equipped with an LC-20A UV-detector and an LC-20 solution data management system (Shimadzu, Duisburg, Germany). In the stationary phase, an XBridge BEH C18 2.5 µm 30 × 4.5 mm analytical column (Waters, Eschborn, Germany) was used, preceded by a Nucleoshell RP18 2.7 m (4 × 3 mm) guard column (Macherey-Nagel, Düren, Germany), the mobile phase consisted of 20 mM sodium phosphate/acetonitrile 68:32 (*v*/*v*), pH 7.1. The column temperature was kept at 40 °C. Samples were directly injected into the HPLC system with an injection volume of 0.5 µL. At a flow rate of 1 mL/min voriconazole eluted at 2.1 min. The detection wavelength was 255 nm. Standard substance voriconazole (PF-00579955, free base, potency 100%) was obtained from Pfizer (Peapack, NJ, USA) and dissolved in DMSO. For this assay, linearity has been proven between 13 mg/mL and 7 mg/mL. Determination of the precision by repeat measurements gave a CV of 0.28%.

#### 2.7.2. Chromatography for Anidulafungin

Anidulafungin concentrations in H_2_O were quantified by high performance liquid chromatography (HPLC) and UV detection as described previously by Welte et al. [9]. Samples were 1:200 diluted to meet the calibration range of 0.01–20.0 µg/mL.

## 3. Results

### 3.1. Minimal Inhibitory Concentration (MIC)

MIC results of clinical isolates and ATCC strains showed no statistical difference (Wilcoxon signed rank test) in the activity between generic and innovator antimycotic in all settings. Additionally, ratios between the originator and the generics for each isolate have been calculated, and all combinations showed an overall median ratio of 1. Thus, we can confirm the interchangeability of the drugs, as seen in Table 1.

### 3.2. Time Kill Curves (TKC)

Time Kill Curves (TKC) and Growth Controls (GC) with voriconazole against ATCC-90028 *C. albicans* and anidulafungin against the clinical isolate *C. glabrata* 722H were done comparing originator and generic drugs.

**Figure 1:**
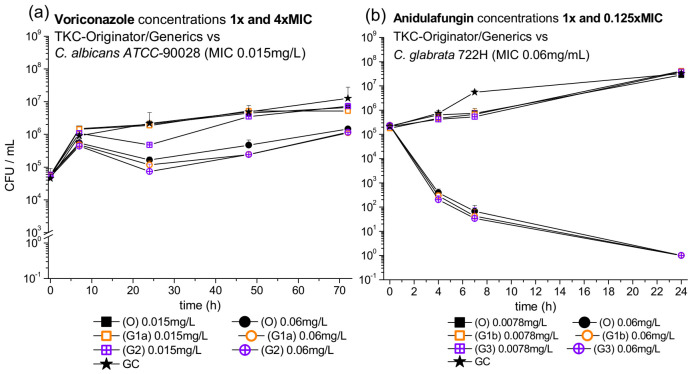
TKC of (**a**) voriconazole against *C. albicans* and (**b**) anidulafungin against *C. glabrata* tested with originators (O) from Pfizer (black symbols), generics–1 (G1a and G1b) from Strada (orange symbols), generic–2 (G2) from Ratiopharm and generic–3 G3 from Pharmore (crossed purple symbols) with standard deviations over 72 and 24 h, respectively, are shown. Additionally, the growth control (GC) is depicted with star symbols.

In Figure 1, CFU/mL over time for voriconazole (a) challenged with concentrations of 1× and 4× MIC and anidulafungin (b) challenged with concentrations of 1× and 0.125× MIC are shown. Results of all other concentrations can be found in Supplementary Figures S1 and S2.

### 3.3. Stability Testing

Stability testing demonstrated the stability of the tested antimycotics despite originator or generic status.

In detail, voriconazole and anidulafungin were stable at 4 °C and room temperature. Measurements of the samples showed that differences from the initial value were below 1%. Thus, the stability of voriconazole over 24 h can be granted for originator and generics.

**Figure 2:**
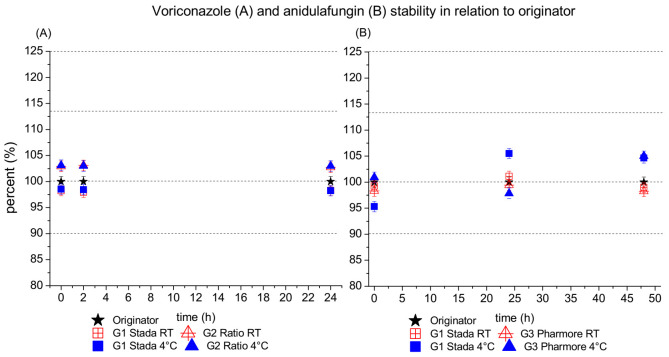
This figure shows the relative stability of the generics of voriconazole (**A**) and anidulafungin (**B**) in relation to the originator (set to 100%), within 48 and 24 h, respectively. Red symbols represent room temperature data (RT), and blue symbols the values at 4 °C. The star symbol represents the originator, set to 100%. The standard deviation is shown for all values but is partly overlaid by symbols. Additionally, the main peak value margins (80%, 90%, 100%, 113% and 125%) are given as dotted lines.

The relative stability of generic antifungals of voriconazole (A) and anidulafungin (B) are presented in Figure 2. Mean values (+/−SD) were calculated by comparing concentrations of generic antifungals to the originator at each investigated time point and temperature condition. Moreover, chromatograms and UV-spectra for voriconazole and anidulafungin of originator and generics are depicted in Supplementary Figures S3 and S4.1–S4.3.

## 4. Discussion

As concerns of the interchangeability between originator and generic have been discussed in different studies, we set out to investigate the in vitro activity and efficacy of antimycotic generics and innovator of voriconazole and anidulafungin against clinical fungal isolates that could confirm interchangeability between the tested drugs.

While the median MIC for voriconazole and anidulafungin against tested strains varied between originator and generics, no relevant or statistical difference was present as the ratios between the originator and the generics (calculated for each isolate, the ATCC-strain and all combinations) also showed an overall median ratio of 1, indicating no difference.

Data from time-killing experiments with the selected strains challenged with voriconazole and anidulafungin strengthen our hypothesis.

Further, antimycotic stability within approved storing conditions could be confirmed, indicating that also in clinical practice there are no differences regarding requirements for storing the clinical preparations.

One particular strength of this study was using a collection of isolates of the most frequent fungal pathogens that would routinely be treated with voriconazole and anidulafungin, meaning that transparent and conclusive results could be obtained. Moreover, stability testing of originator and generics has been conducted, securing comparable conditions within the test settings.

Additionally, it was assured that generics and originator substances used in this study were produced at a different manufacturing site. This seems of particular relevance since many “generic brands” actually originate from the same factory, which would make a comparison meaningless.

A limitation of the study might be that only an excerpt of 2 generics for each setting has been tested (e.g., for voriconazole, further generics exist from Hikma and Accord [10,11]).

Moreover, tests were conducted using one batch only, and thus differences between product batches might not be detected.

Furthermore, most in vitro tests were performed with only one species for each drug, and therefore, potential inter-species differences might not be detected (e.g., *Candida* vs. *Aspergillus*). Naturally, this study faces the limitations of an in vitro study, and this limited space and nutrition must be considered.

A further approach to confirming the interchangeability of generic and originator might be bioequivalence (in healthy volunteers) or efficacy studies in-vivo (in animal experiments or patients). Nevertheless, even though animal studies would indeed determine the antifungal efficacy, based on the current data we do not believe they would result in additional insights, and we decided not to perform animal studies in consideration of animal welfare.

In conclusion, our data show the in vitro interchangeability of generics and innovators, which might lead to a broader public acceptance of generic antimycotics.

## Figures and Tables

**Table 1:** Median MIC given in mg/L of clinical isolates and ATCC-strains is shown for originator and generic. The manufacturing companies are depicted in the table as followed; Pfizer-originator (O); Stada-generic_1 (G1); Ratiopharm-generic_2 (G2); Pharmore-generic_3 (G3). Additionally, median values of the ratios of originator and generic are presented. * No significant difference could be shown with the Wilcoxon signed rank test.

Setting	Average MIC in mg/L			Median Value of the Ratio of Each Isolate	
**Voriconazole**	**(O)**	**(G1)**	**(G2)**	**O/G1**	**O/G2**
**21 clinical isolates *C. albicans***	0.0073	0.0071	0.0068	1 *	1 *
**ATCC-90028**	0.065	0.065	0.065	1 *	1 *
**Anidulafungin**	**(O)**	**(G1)**	**(G3)**	**O/G1**	**O/G3**
**20 clinical isolates** ***C. glabrata***	0.06	0.06	0.06	1*	1 *
**ATCC-22019**	0.5	0.5	0.5	1 *	1 *

## Data Availability

Not applicable.

## Supplementary Materials

The following are available online at www.mdpi.com/article/10.3390/jof8020195/s1, Figure S1: TKC of voriconazole against *C. albicans* tested with originator, generic-1 and generic-2. Figure S2: TKC of anidulafungin against *C. glabrata* tested with originator, generic-1 and generic-2. Figure S3: Chromatogram of voriconazole with originator, generic 1 and generic 2; Figure S4: Chromatogram of anidulafungin with originator, generic 1 and generic 2.
