# Supplementary material for: Comparison of Antimycotic Activity of Originator and Generics of Voriconazole and Anidulafungin against Clinical Isolates of Candida albicans and Candida glabrata
Source: J Fungi (Basel). 2022 Feb 17;8(2):195. doi: 10.3390/jof8020195 (PMC8879245; doi:10.3390/jof8020195)
Supplement: Supplementary file 1 [file jof-08-00195-s001.zip › Supplement-Fungi-Generics-MDPI-Fungi-S1 and S2.pdf]

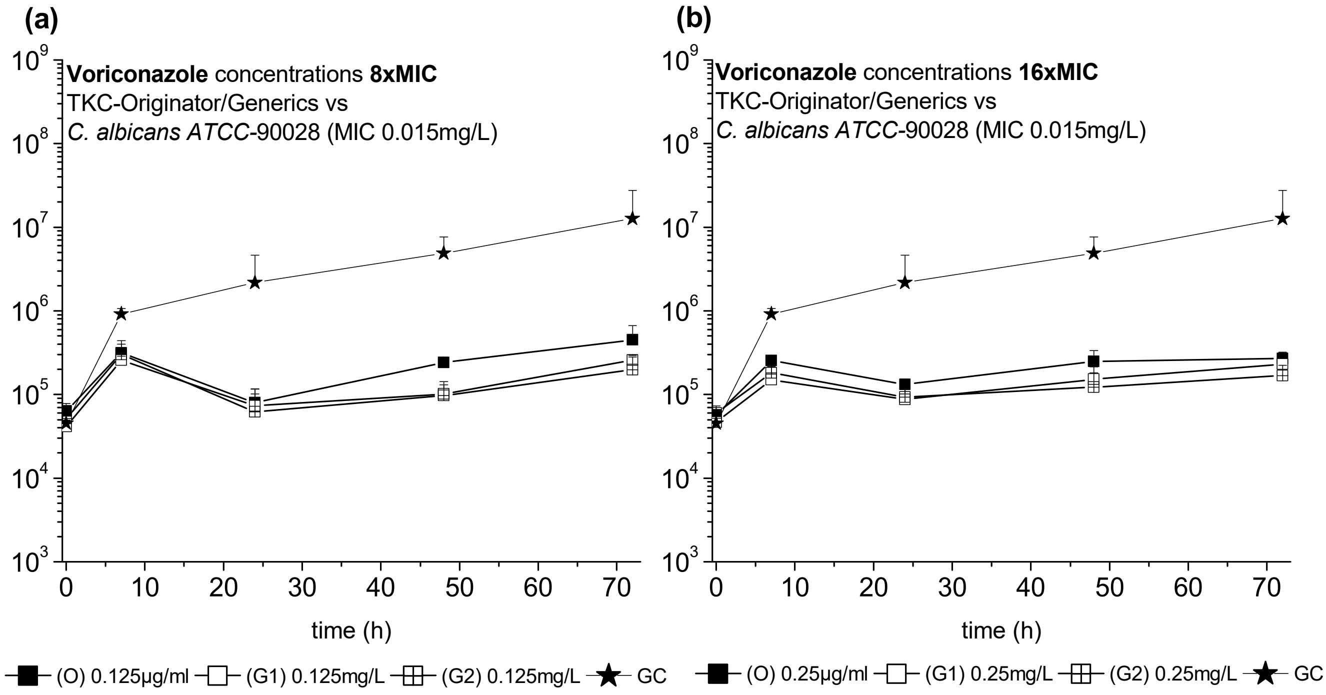

Figure S1: TKC of voriconazole against *C. albicans* tested with **originator (O)** from Pfizer **(black symbols)**, **generic-1 (G1)** from Strada **(white symbols)**, **generic-2 (G2)** from Ratiopharm **(crossed symbols)** with concentrations 0.125mg/L **(a)** and 0.25mg/L **(b)** over 72 hours are shown. Standard deviations are given. Additionally, the **growth control (GC)** is depicted with **star symbols**.

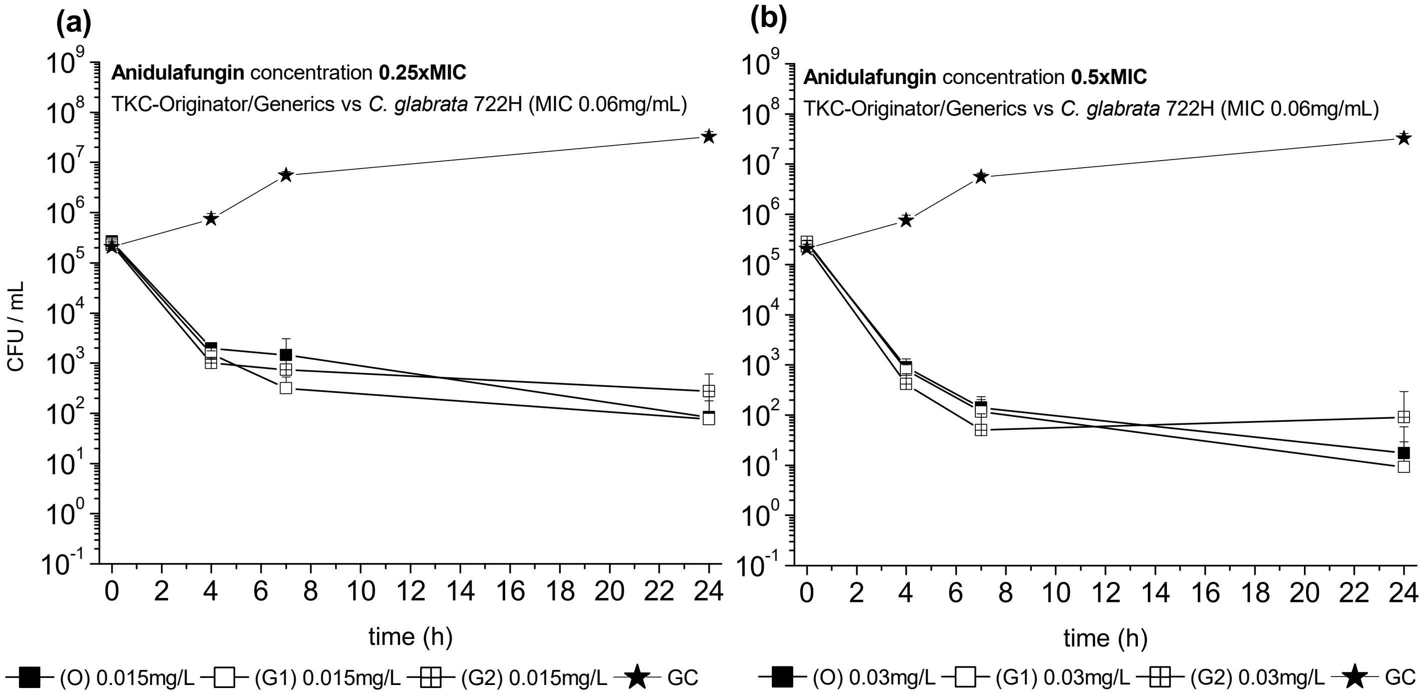

Figure S2: TKC of anidulafungin against *C. glabrata* tested with **originator (O)** from Pfizer **(black symbols)**, **generic-1 (G1)** from Strada **(white symbols)**, **generic-2 (G2)** from Pharmore **(crossed symbols)** with concentrations 0.015mg/L **(a)** and 0.03mg/L **(b)** over 24 hours are shown. Standard deviations are given. Additionally, the **growth control (GC)** is depicted with **star symbols**.
