# Supplementary material for: Comparison of Antimycotic Activity of Originator and Generics of Voriconazole and Anidulafungin against Clinical Isolates of Candida albicans and Candida glabrata
Source: J Fungi (Basel). 2022 Feb 17;8(2):195. doi: 10.3390/jof8020195 (PMC8879245; doi:10.3390/jof8020195)
Supplement: Supplementary file 1 [file jof-08-00195-s001.zip › Supplement-Fungi-Generics-MDPI-Fungi-S3.pdf]

A

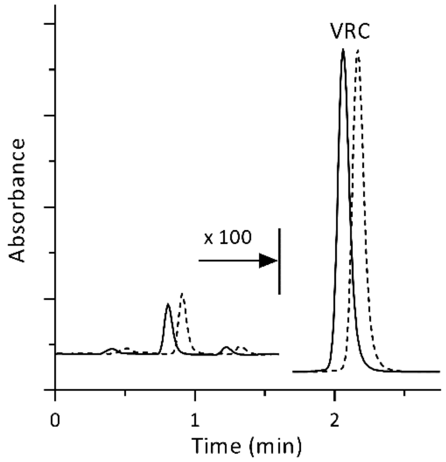

B

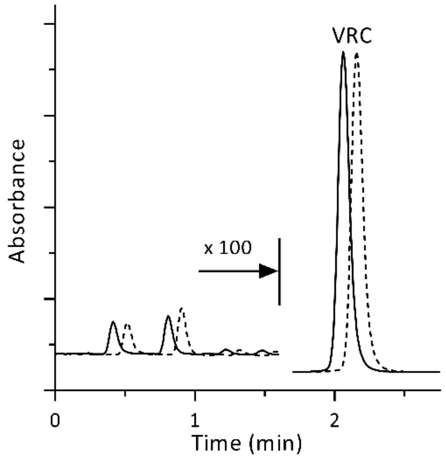

C

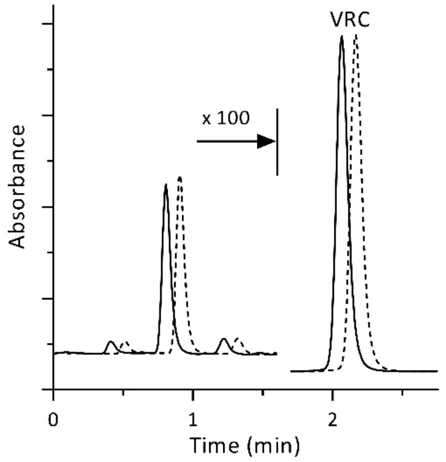

Suppl. Figure S3: Chromatograms of voriconazole (VRC) in aqueous solutions of (A) VFEND® from Pfizer and generics from (B) Stada and (C) Ratiopharm at baseline (solid lines) and after 24 h of storage at room temperature (dashed lines). The chromatographic signal has been amplified by factor 100 until 1.7 minutes to visualize peaks deriving from (nonquantifiable) traces of unknown by-products (content <1%).
