# Supplementary material for: Comparison of Antimycotic Activity of Originator and Generics of Voriconazole and Anidulafungin against Clinical Isolates of Candida albicans and Candida glabrata
Source: J Fungi (Basel). 2022 Feb 17;8(2):195. doi: 10.3390/jof8020195 (PMC8879245; doi:10.3390/jof8020195)
Supplement: Supplementary file 1 [file jof-08-00195-s001.zip › Supplement-Fungi-Generics-MDPI-Fungi-S4.2.pdf]

(A)

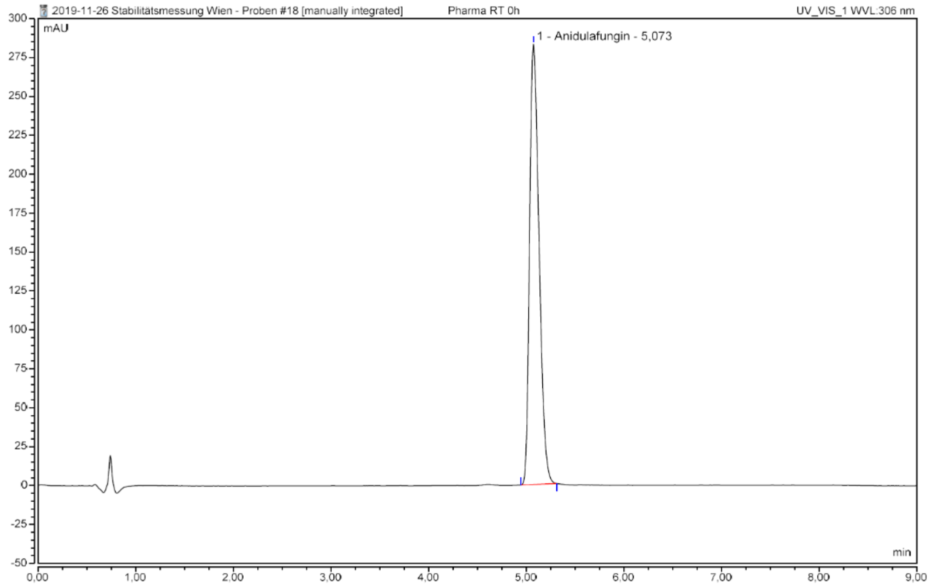

(B)

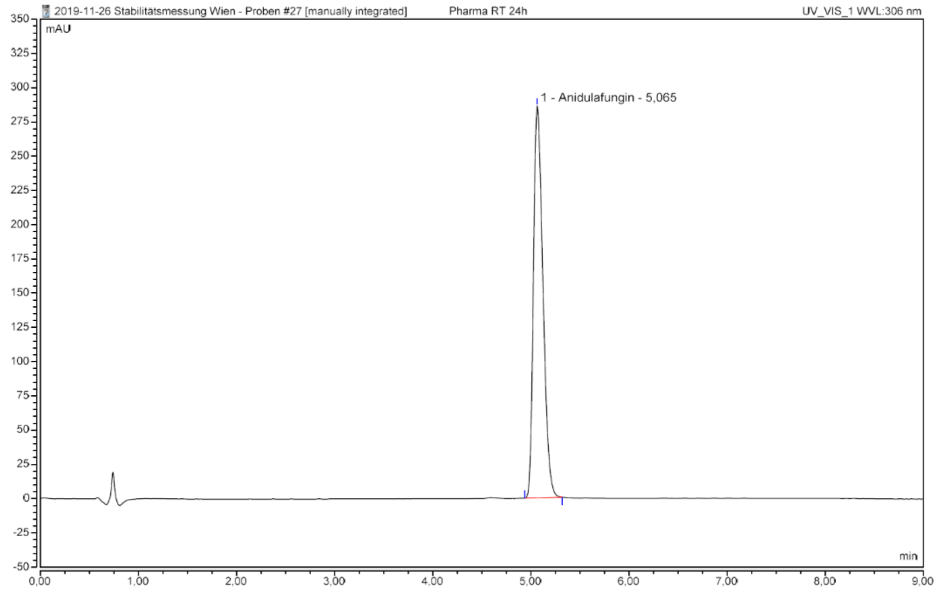

(C)

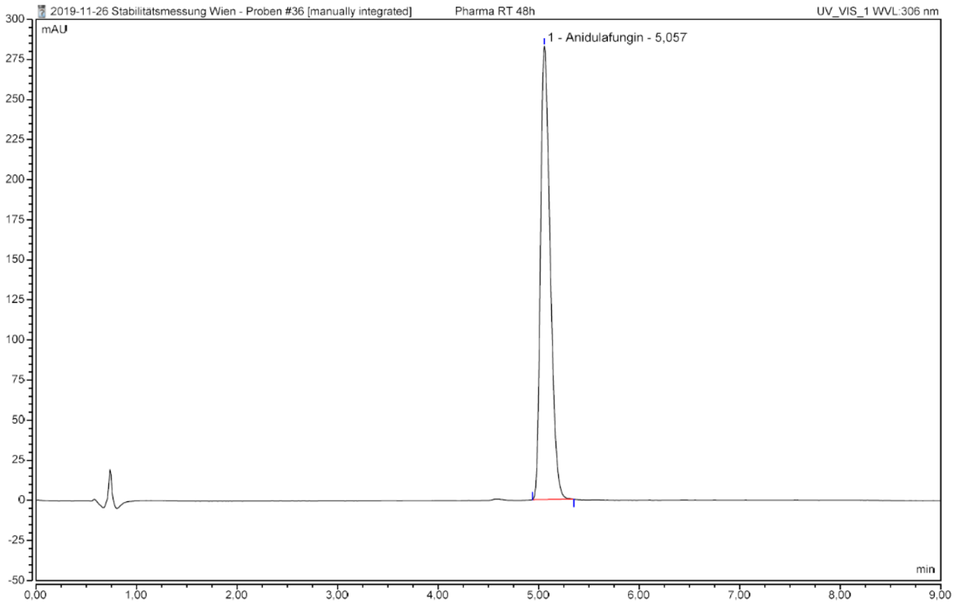

Suppl. Figure S4.2: Chromatograms of anidulafungin in aqueous solutions from Pharma at (A) baseline, (B) after 24 h and (C) after 48h of storage at room temperature. The small peak shown is derived from (nonquantifiable) traces of unknown by-products (content <1%).
